# Mitochondria-Targeted Antioxidant SkQ1 Improves Dermal Wound Healing in Genetically Diabetic Mice

**DOI:** 10.1155/2017/6408278

**Published:** 2017-07-06

**Authors:** Ilya A. Demyanenko, Vlada V. Zakharova, Olga P. Ilyinskaya, Tamara V. Vasilieva, Artem V. Fedorov, Vasily N. Manskikh, Roman A. Zinovkin, Olga Yu Pletjushkina, Boris V. Chernyak, Vladimir P. Skulachev, Ekaterina N. Popova

**Affiliations:** ^1^Faculty of Biology, Lomonosov Moscow State University, Leninskie Gory 1-12, Moscow 119234, Russia; ^2^Belozersky Institute of Physico-Chemical Biology, Lomonosov Moscow State University, Leninskie Gory 1-40, Moscow 119992, Russia; ^3^Institute of Mitoengineering, Lomonosov Moscow State University, Leninskie Gory 1-73, Moscow 119992, Russia

## Abstract

Oxidative stress is widely recognized as an important factor in the delayed wound healing in diabetes. However, the role of mitochondrial reactive oxygen species in this process is unknown. It was assumed that mitochondrial reactive oxygen species are involved in many wound-healing processes in both diabetic humans and animals. We have applied the mitochondria-targeted antioxidant 10-(6′-plastoquinonyl)decyltriphenylphosphonium (SkQ1) to explore the role of mitochondrial reactive oxygen species in the wound healing of genetically diabetic mice. Healing of full-thickness excisional dermal wounds in diabetic C57BL/KsJ-db^−^/db^−^ mice was significantly enhanced after long-term (12 weeks) administration of SkQ1. SkQ1 accelerated wound closure and stimulated epithelization, granulation tissue formation, and vascularization. On the 7th day after wounding, SkQ1 treatment increased the number of *α*-smooth muscle actin-positive cells (myofibroblasts), reduced the number of neutrophils, and increased macrophage infiltration. SkQ1 lowered lipid peroxidation level but did not change the level of the circulatory IL-6 and TNF. SkQ1 pretreatment also stimulated cell migration in a scratch-wound assay in vitro under hyperglycemic condition. Thus, a mitochondria-targeted antioxidant normalized both inflammatory and regenerative phases of wound healing in diabetic mice. Our results pointed to nearly all the major steps of wound healing as the target of excessive mitochondrial reactive oxygen species production in type II diabetes.

## 1. Introduction

Impaired wound healing and chronic wounds are a significant source of complications of diabetes mellitus. Wound healing is a complex sequence of cellular and molecular processes consisting of inflammation, formation of the granulation tissue (including myofibroblast accumulation, extracellular matrix synthesis, and angiogenesis), reepithelialization, and tissue remodeling. The impact of diabetes is widespread and pleiotropic, affecting the majority of cells and mechanisms involved in the repair process. These in particular include prolonged and exacerbated inflammatory stage, inadequate expression of growth factors at the site of injury, impaired angiogenesis, dysfunction of fibroblasts and epidermal cells, and impaired ability of bone marrow progenitor cells to migrate to the lesion [1].

Oxidative stress is now recognized as a key participant in the development of many diabetic complications. Excessive generation of reactive oxygen species (ROS) in diabetes is largely due to acute rises in serum glucose and accumulation of advanced glycation end products (AGEs) [2]. A delicate balance between the positive role of ROS and their deleterious effects is important for proper wound healing. Oxidative stress in diabetes may be involved in nearly all of the above-mentioned pathologies related to impaired wound healing [1, 3, 4].

It was hypothesized that the damaging effects of hyperglycemia are activated by mitochondrial ROS (mtROS) overproduction [5, 6]. However, a recently proposed mitochondrial hormesis theory challenged this point of view [7]. Probably, mtROS are also the key players in the development of insulin resistance [8] thus participating in a vicious cycle of diabetes progression.

Plastoquinone derivatives covalently conjugated with lipophilic cations (SkQ) are one of the most intensively studied groups of mitochondria-targeted antioxidants [9]. It was shown that SkQ1 treatment of obese mice kept on a high-fat, high-sucrose diet normalized H_2_O_2_ and protein carbonyl levels in muscles [10]. At the same time, SkQ1 did not affect weight gain, triacylglycerol, glucose, and insulin level in plasma and did not protect insulin signaling in the skeletal muscle. Interestingly, SkQ1 prevents high-fat diet-induced increases in mitochondrial biogenesis probably mediated by activation of Ca^2+^/calmodulin-dependent protein kinase CaMKII [10, 11].

In our recent study, we have found that long-term (8 months) oral administration of SkQ1 dramatically improves impaired dermal wound healing in aging mice [12]. In the present study, we have investigated whether SkQ1 could affect wound healing in the mice model of type II diabetes.

## 2. Materials and Methods

### 2.1. Animals and Antioxidant Treatment

Male mutant homozygous C57BL/KsJ-db^−^/db^−^ (*db*/*db*) mice were obtained from the Center of Biomedical Technologies, RAMS, Moscow. The experimental group of animals (*n* = 12) received 250 nmol/kg of body weight per day of SkQ1 (synthesized at Institute of Mitoengineering, Lomonosov Moscow State University) per os for 12 weeks starting from the 9th week of life. The daily dose of SkQ1 was chosen according to our earlier studies [9]. The control group of *db*/*db* mice (*n* = 9) and heterozygous C57BL/KsJ-db^+^/db^−^ (*db*/+) mice (*n* = 5) was treated with a vehicle (20% glycerol). Heterozygous mice had normal phenotype and did not display any diabetic features.

### 2.2. Ethical Approval

All animal care and experimental procedures complied with Guide for the Care and Use of Laboratory Animals, Eighth edition (2011).

### 2.3. Blood Glucose, Glycated Hemoglobin, and Body Mass Analysis

During administration of the SkQ1, body mass and blood glucose levels were measured every two-three weeks. In the final week of treatment, the level of glycated hemoglobin (HbA1c) was also analyzed. For all measurements, blood was collected from the tail vein (5 *μ*l) after 12 hours of starvation. Blood glucose and glycated hemoglobin were measured with an Accu-Check Perfoma Nano (Roche Diagnostics, France) and NycoCard Reader II (Axis-Shiel, Norway), respectively.

### 2.4. Wounding Protocol and Wound Closure Analysis

Animals were anesthetized using zoletil (50 mg per 1 kg of body weight). Hair on the dorsal side of mice was shaved and the skin was cleaned with 70% ethanol. Full-thickness excisional skin wound 0.7 cm in diameter was created surgically in the interscapular area. No dressing was placed on the wounds.

For macroscopic examination, digital photos of the wound area were made once a day. The wound surface area was measured at the photographs using ImageJ software.

### 2.5. Blood Cytokine Level and Liver TBARS Measurement

At the 7th day of healing, animals were euthanized by decapitation, blood was collected, and the serum concentrations of IL-6 and tumor necrosis factor (TNF) were determined with enzyme-linked immunosorbent assay (ELISA) kits (eBioscience, USA) according to manufacturer's protocols. Liver samples were excised, and thiobarbituric acid reactive substance (TBARS) content was measured by the method of Mihara and Uchiyama [13].

### 2.6. Histological and Immunohistochemical Studies

After euthanasia, wounds with surrounding tissues were excised and fixed with 10% phosphate-buffered (pH = 7.4) formalin. The samples were transversely cut exactly through the center of the wound, dehydrated, and paraffin-embedded according to the routine protocols. Cross sections (5 *μ*m thick) were stained with hematoxylin and eosin (H&E) and Mallory's trichrome stain. For immunostaining, sections were treated with 3% H_2_O_2_ for 10 min and then with 10% nonimmune goat serum before incubation with rabbit polyclonal antibodies against *α*-smooth muscle actin (*α*-SMA) and CD31 (Abcam, UK) or rat monoclonal antibodies against f4/80 (Serotec, UK). Goat anti-rabbit and anti-rat IgG biotinylated antibodies (Vector, USA) were applied and stained with avidin-peroxidase conjugate and diaminobenzidine (Vector, USA). Before CD31 staining, heat-mediated antigen retrieval with Vector Antigen Unmasking Solution (Vector, USA) at 98°C for 40 min was applied additionally.

Samples were analyzed with a DM 5000B microscope equipped with a DFC 320 digital camera (Leica, Germany).

### 2.7. Morphometric Analysis

The granulation tissue area and epithelization of wound were analyzed on the H&E-stained sections. Epithelization was calculated as the ratio of the wound surface covered by regenerating epidermis to the total wound surface. Vessel density was calculated as the ratio of the summarized blood vessel area revealed with CD31 immunostaining to the granulation tissue area. Neutrophils were counted on H&E-stained sections and macrophages were detected as f4/80-positive cells after immunostaining. Histomorphometric analysis was performed on digital microphotographs by ImageJ software.

Myofibroblasts areal density was measured after immunostaining as the ratio of *α*-SMA-positive cytoplasm to the total area of granulation tissue. A method of color subtractive-computer-assisted image analysis [14] was applied.

### 2.8. In Vitro Studies of Fibroblast Motility

Human subcutaneous fibroblasts HSF (Russian Cell Culture Collection, Institute of Medical Genetics, Russian Academy of Sciences) were grown in Dulbecco's modified Eagle's medium (DMEM) (Gibco, USA) supplemented with 10% fetal calf serum (Hyclone, USA) at 37°C in a humidified atmosphere containing 5% CO_2_. Cells were incubated with 20 nM SkQ1 for 3 days and transferred to the fresh medium with 25 mM glucose (control), 50 mM glucose (hyperglycemia), or 50 mM mannitol (osmotic control) with 20 nM SkQ1. After 24 hours, the cells were scratched with the rile pipette tip. Cell motility was analyzed in a scratch-wound assay as described earlier [12]. The images were taken with an Axiovert microscope (Carl Zeiss, Germany) equipped with an Axiocam camera (Carl Zeiss), and “wound” areas were analyzed using ImageJ software.

### 2.9. Statistical Analysis

Statistical analysis was done with STATISTICA 7.0 software. The data were expressed as mean ± SEM or mean ± SD (see figure legend). Mann–Whitney *U* test or Kruskal-Wallis *H* test (one-way ANOVA on ranks) followed by Dunn's test for multiple comparisons were conducted, and significance was set at level *P* < 0.05.

## 3. Results

**Figure 1:**
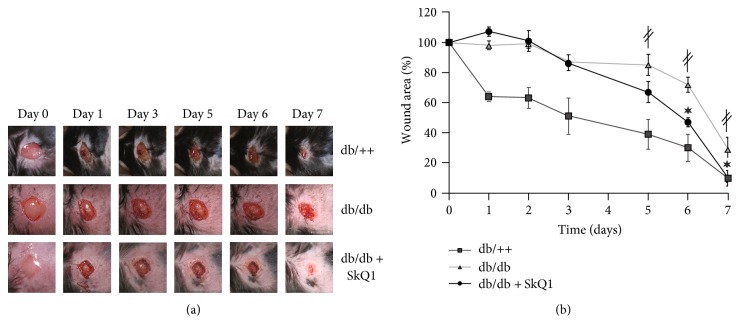
SkQ1 accelerates wound closure in diabetic mice. Full-thickness excisional skin wounds were created surgically in the interscapular area on the back of nondiabetic (db/++, *n* = 5), diabetic (db/db, *n* = 9), and diabetic mice receiving SkQ1 (250 nmol/kg of body mass per day) during 12 weeks (*n* = 12). (a) Representative images of the wounds, (b) dynamics of the wound closure. Data are presented as mean ± SEM; ^∗^*P* < 0.05 for SkQ1-treated versus untreated diabetic mice. ^*‡*^*P* < 0.05 for the untreated diabetic mice versus nondiabetic mice.

**Figure 2:**
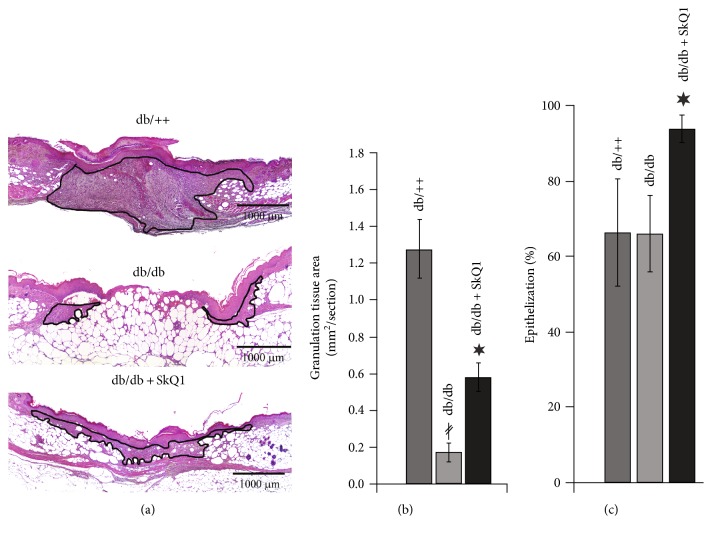
SkQ1 promotes granulation tissue formation and epithelization of the wounds in diabetic mice. (a) Representative images of the H&E-stained transverse sections of the wounds at the 7th day of healing. (b) Granulation tissue formation and (c) epithelization of the wounds. Data are presented as mean ± SEM; ^∗^*P* < 0.05 for SkQ1-treated versus untreated diabetic mice. ^*‡*^*P* < 0.05 for the untreated diabetic mice versus nondiabetic mice.

**Figure 3:**
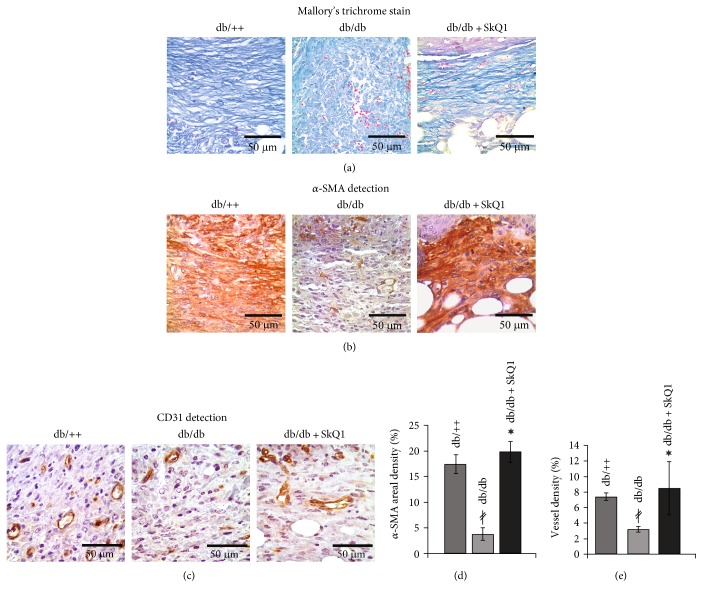
Effect of SkQ1 on the maturation of granulation tissue in diabetic mice. Representative images of the (a) Mallory's trichrome-stained and (b) ɑ-SMA- and (c) CD-31-immunostained granulation tissue at the 7th day of wound healing. (d) Percentage of the area containing *α*-SMA-positive cytoplasm (areal density). (e) Percentage of the area containing microvessels (vessel density). Data are presented as mean ± SEM; ^∗^*P* < 0.05 for SkQ1-treated versus untreated diabetic mice. ^*‡*^*P* < 0.05 for the untreated diabetic mice versus nondiabetic mice.

Comparative analysis of the wound healing in *db*/*db* versus *db*/+ mice has shown a dramatic delay in wound area contraction (Figure 1) and granulation tissue formation (Figures 2(a) and 2(b)). However, epithelization of the wounds was not impaired (Figure 2(c)). These observations are in a perfect agreement with the studies pointed to *db*/*db* mice as a good experimental model of type II diabetes-impaired wound healing [15, 16]. Oral administration of 250 nmol/kg SkQ1 resulted in a significant reduction of wound area in *db*/*db* mice on the 6th and 7th days, so on the 7th day, the wound size was the same as in *db*/++ mice (Figure 1(b)). Administration of SkQ1 also strongly increased the amount of granulation tissue in diabetic animals (Figures 2(a) and 2(b)). Moreover, new-formed connective tissue of SkQ1-treated *db*/*db* mice consisted of more mature and regularly oriented bundles of collagen fibers compared to the granulation tissue of control animals (Figures 2(a) and 2(b) and Figure 3(a)). SkQ1 induced the dramatic increase in content of *α*-SMA-positive fibroblast-like cells referred to as myofibroblasts (Figures 3(b) and 3(d)). These cells play an important role in the formation and maturation of the granulation tissue due to increased formation of extracellular matrix molecules (including collagen) and growth factors. Furthermore, myofibroblasts can directly participate in mechanical wound closure due to their contractility.

Treatment with SkQ1 significantly stimulated epithelization of the wounds in diabetic animals (Figure 2(c)) though it was not compromised in *db*/*db* mice in line with earlier observations [16].

Diabetes mellitus is often accompanied by macro- and microvascular complications leading to local tissue hypoxia and chronic wound formation. Vascularization of the wounds was delayed in *db*/*db* mice (Figure 3(c)). Treatment with SkQ1 significantly increased vessel density in granulation tissue (Figures 3(c) and 3(e)). This effect probably contributes to the acceleration of tissue regeneration.

**Figure 4:**
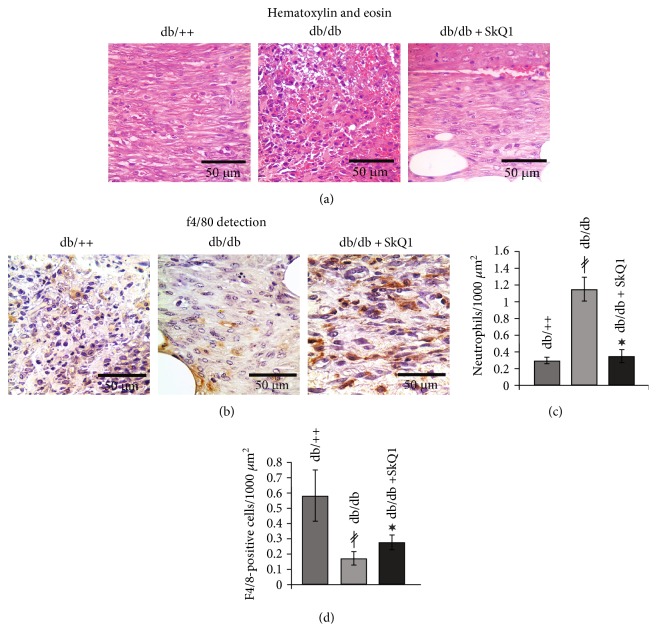
Effect of SkQ1 on the leukocyte composition of granulation tissue in diabetic mice. Representative images of the (a) H&E-stained and (b) f4/80-immunostained granulation tissue at the 7th day of wound healing. (c) Neutrophil and (d) macrophage (F4/80-positive cells) infiltration of the granulation tissue. Data are presented as mean ± SEM; ^∗^*P* < 0.05 for SkQ1-treated versus untreated diabetic mice. ^*‡*^*P* < 0.05 for the untreated diabetic mice versus nondiabetic mice.

**Figure 5:**
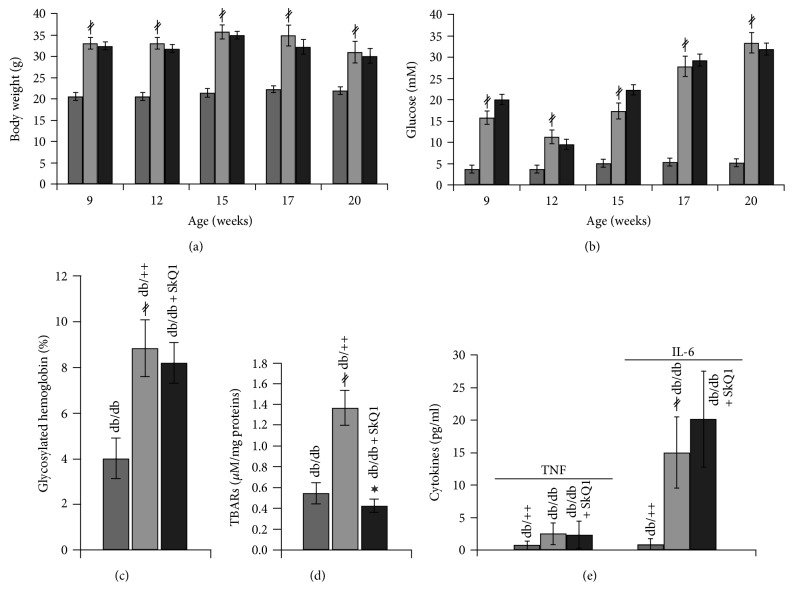
Effect of SkQ1 on the metabolic parameters and circulatory cytokine levels of diabetic mice. Dynamics of (a) body mass and (b) blood glucose level during the antioxidant administration. (c) Glycated hemoglobin level at the 11th week of treatment. Data are presented as mean ± SEM. (d) Liver TBARS level and (e) serum cytokine concentration at the 7th day of wound healing. Data are presented as mean ± SD. ^∗^*P* < 0.05 for SkQ1-treated versus untreated diabetic mice. ^*‡*^*P* < 0.05 for the untreated diabetic mice versus nondiabetic mice.

Persistence of neutrophil infiltration and a delay in accumulation of macrophages were observed in the wounds of *db*/*db* mice (Figure 4), indicating the delay in the resolution of an inflammatory phase of wound healing. Administration of SkQ1 decreased the number of neutrophils in the granulation tissue of *db*/*db* mice to the level observed in their nondiabetic *db*/++ littermates (Figures 4(a) and 4(c)) and significantly increased macrophage content (Figures 4(b) and 4(d)). The effect of SkQ1 on the neutrophil and macrophage infiltration was not related to the changes of inflammatory status in *db*/*db* mice. We have detected significant elevation in the level of proinflammatory cytokines TNF and IL-6 in plasma of diabetic mice with strong individual variations of these parameters. Treatment with SkQ1 did not change the level of these cytokines (Figure 5(e)). We suggest that SkQ1 accelerated the resolution of an inflammation phase of wound healing by local inhibition of inflammatory activation of endothelial cells and by improvement of immune cells functioning in the regenerating tissues, rather than by a systemic anti-inflammatory effect.

SkQ1 affected neither serum glucose level nor weight gain in diabetic mice (Figures 5(a) and 5(b)). The level of glycated HbA1c reflecting sustained hyperglycemia was also not affected (Figure 5(c)). At the same time, SkQ1 significantly decreased the level of lipid peroxidation end products (measured as TBARS) in the liver of *db*/*db* mice confirming high antioxidant efficiency of this compound (Figure 5(d)).

**Figure 6:**
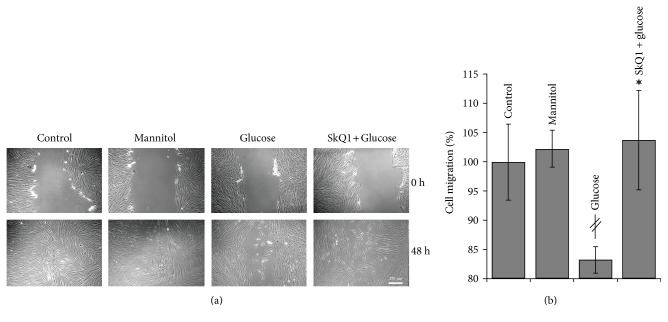
Effect of SkQ1 on the cell motility in the scratch-wound assay under high-glucose conditions. Migration of human subcutaneous fibroblasts in a scratch-wound assay (48 h after wounding). (a) Representative images. (b) Percentage of the wound area occupied with migrated cells. “Control”—control medium (25 mM glucose); “mannitol”—control medium (50 mM mannitol); “glucose”—high-glucose medium (50 mM glucose); “SkQ1 + glucose”—high-glucose medium (20 nM SkQ1). Data are presented as mean ± SEM; ^*‡*^*P* < 0.05 for the high-glucose versus control medium. ^∗^*P* < 0.05 for SkQ1-treated high-glucose versus high-glucose medium.

We have shown earlier that SkQ1 initiated myofibroblast differentiation and actin cytoskeleton rearrangements in fibroblasts in vitro [17]. These changes were accompanied by the increased migration of fibroblasts into the scratched “wound” made in a cell monolayer [18]. Migration of fibroblasts was strongly inhibited by high-glucose (50 mM) medium and SkQ1 pretreatment prevented this inhibition (Figure 6). These data indicate that fibroblasts could be directly affected by excessive mtROS production caused by hyperglycemia both in vivo and in vitro.

## 4. Discussion

Our study demonstrated that mitochondria-targeted antioxidant SkQ1 did not affect weight gain and hyperglycemia in diabetic *db*/*db* mice but suppressed oxidative stress and improved healing of full-thickness excisional skin wounds. These data are in agreement with the results of earlier studies on SkQ1 [10] and on related mitochondria-targeted antioxidant 10-(6′-ubiquinonyl)decyltriphenylphosphonium (MitoQ) [19] that only slightly inhibited development of hyperglycemia and insulin resistance but strongly protected against various complications in animal models of diabetes. These data indicate that excessive mtROS production is crucial for hyperglycemia-induced damage in various tissues being less important for insulin resistance.

Compromised wound healing in diabetes obviously has complex pleiotropic etiology [1], and our results pointed to the major steps of wound healing as the targets of excessive mtROS. Recruitment of neutrophils to the wound is important for controlling microbial invasion, but neutrophil persistence results in the delayed resolution of an inflammatory phase and impairment of diabetic wound healing [20]. In neutropenic db/db mice, wound closure was improved due to accelerated reepithelialization [21]. The similar effect of SkQ1 on acute wound healing or aseptic inflammation was observed earlier in healthy animals [18].

ROS may enhance insulin action [22, 23] and insulin secretion [24, 25]. On the other hand, chronic oxidative stress is a central mechanism for glucose toxicity in pancreatic islet beta cells in diabetes [26], and ROS levels are an important trigger for insulin resistance in numerous settings [8]. Thus, theoretically antioxidant treatment could affect the diabetes progression. However, both in our work and in the previous paper, SkQ1 had antioxidant activities but had no effect on the diabetic features [10]. Noteworthy, SkQ1 mostly affected cellular content in the wounds thus implying its potential to be used in the form of topical application. In our previous work, we have observed some stimulatory effect of topical SkQ1 application on the wound healing in young rats [18].

It was previously shown that SkQ1 attenuates expression of adhesion molecules ICAM1, VCAM, and E-selectin in endothelial cell culture treated with TNF as well in aorta of aging mice [27] via inhibition of NF-*κ*B signaling. SkQ1 also prevented TNF-induced decomposition of VE-cadherin-containing contacts, following an increase in the permeability of endothelial cell monolayer [12]. SkQ1 also inhibited apoptosis of endothelial cells induced by high doses of TNF [28]. SkQ1 did not influence the elevated level of proinflammatory cytokines in the blood of diabetic (this study) or aged mice [27], so we suggest that its anti-inflammatory action in the skin wounds of diabetic mice may be at least partially mediated by prevention of endothelial cell activation, following excessive neutrophil infiltration.

Treatment with SkQ1 improved the resolution of an inflammatory phase of wound healing, simultaneously decreasing content of neutrophils and increasing content of macrophages (Figure 4). Macrophages are actively involved in the resolution of inflammation by efficient dead cell clearance (efferocytosis) followed by a transition from pro- to anti-inflammatory prohealing phenotype. A significant delay in macrophage infiltration is typical for diabetic mice [29]. It is followed by increased apoptotic cell burden and a prolonged inflammatory phase [30]. The persistence of the proinflammatory macrophages in the diabetic wounds is mediated by the sustained NLRP3 inflammasome activity [31]. NLRP3 inflammasome activation is dependent on the mtROS production [32], and mitochondria-targeted ROS scavenger MitoQ decreases NLRP3-dependent IL-1 beta and IL-18 production in human macrophage-like cell line THP-1 [33]. It seems reasonable to suggest the similar mechanism for SkQ1 during diabetic wound healing. Currently, targeting of the inflammasome is considered a clinically efficacious strategy in restoring insulin action in humans with type 2 diabetes.

Our results indicated that SkQ1 stimulated growth and maturation of granulation tissue in dermal wounds of diabetic mice (Figure 2). Earlier, it was shown that scavenging of mtROS by SkQ1 in the subcutaneous fibroblast culture stimulated metalloprotease-dependent activation of latent TGF*β*1 and downstream SMAD-dependent expression of EDA isoform of fibronectin and *α*-SMA, the major markers of myofibroblasts [17]. The myofibroblasts produce various growth factors (including TGF*β*1), collagen, and other ECM components and participate in wound contraction due to their contractility.

We have shown earlier that SkQ1 activated the Rho/ROCK/LIMK pathway leading to stabilization of actin filaments and accelerated fibroblast migration into the “wound” in the cell monolayer in vitro [17, 18]. Hyperglycemia inhibited fibroblast migration in vitro and SkQ1 prevented this effect (Figure 6). It was suggested that inhibition of fibroblast motility resulted from impaired cell polarity, protrusion destabilization, and inhibition of adhesion maturation at least in part due to oxidative stress that stimulated Rac1 activity [34]. Our data indicated that hyperglycemia-induced Rac1 activation could be mediated by mtROS either directly or via stimulation of RhoA that balanced Rac1-dependent cytoskeleton rearrangements. The effect of SkQ1 on fibroblast motility at high glucose could be important for stimulation of their migration to the wound area from the surrounding tissues and for granulation tissue formation.

Another potential source of myofibroblasts is multipotent mesenchymal stromal cells (MSC) from the bone marrow [35]. Diabetes is accompanied by the bone marrow dysfunction due to neuropathy and microangiopathy leading to the niche dysfunction [36]. p66shc protein is known to stimulate mtROS production. Its knockout partially rescued the defective progenitor cell mobilization from the bone marrow in two different mice models of diabetes [37], so the damaging role of mtROS could be suggested [38]. In agreement with this suggestion, we have found earlier that SkQ1 strongly promoted accumulation of MSC progeny fibroblast colony-forming units (CFU-F). CFU-F content was doubled after SkQ1 treatment in the bone marrow of mice [39]. This source of myofibroblasts may also be important for granulation tissue formation in diabetic mice.

Earlier, we have shown that SkQ1 stimulated active TGF*β*1 secretion by fibroblasts [17]. TGF*β*1 is known for decades as an important regulator of wound healing deficient in diabetic wounds [40]. TGF*β*1 also promotes macrophage differentiation into alternatively activated (or M2) macrophages capable of active efferocytosis [41], and this effect could be implicated in the resolution of inflammation in addition to the effects of mtROS scavenging on neutrophil infiltration and inflammasome inhibition (see above). Moreover, growth medium conditioned with SkQ1-treated fibroblasts stimulated endothelial cell migration and tubular structure formation in vitro in a matrigel angiogenesis assay [12]. Stimulation was at least partially mediated by TGF*β*1 since the inhibitor of its receptor TGF*β*1R1 suppressed the effect [12]. Increased secretion of TGF*β*1 (and probably some other growth factors) by fibroblasts could be responsible for improvement of angiogenesis in diabetic wounds (Figure 3).

The other target of SkQ1 treatment could be endothelial progenitor cells (EPCs), a key cell type involved in angiogenesis. It was found that the number of circulating EPCs and ex vivo functions of EPCs were impaired in diabetic patients due to oxidative stress [3]. MtROS produced in a p66shc-dependent manner in the bone marrow were shown to determine defective EPC mobilization in diabetic mice [37]. Moreover, transplantation of diabetic EPCs after MnSOD gene therapy restored their ability to mediate angiogenesis and wound repair [42], indicating the key role of mtROS in EPC dysfunction.

We have previously shown that long-term (8 months) administration of SkQ1 prevented age-dependent impairment of dermal wound healing in mice [12]. In the present study, SkQ1 treatment improved the same components of a wound healing process in genetically diabetic mice. These findings are in line with numerous studies demonstrating therapeutic action of mitochondria-targeted antioxidants against various age-related disorders [9, 12, 43, 44] and support the key role of mtROS in pathogenesis of many complications inherent to both aging and diabetes.

## 5. Conclusions

The mitochondria-targeted antioxidant SkQ1 enhances dermal wound healing in diabetic mice accompanied by improved resolution of inflammation, increased myofibroblast content, and vascularization of granulation tissue. These results imply that nearly all major stages of diabetic wound healing are hindered by excessive mitochondrial reactive oxygen species production.
